# The Effects of Glucocorticoid and Voluntary Exercise Treatment on the Development of Thoracolumbar Kyphosis in Dystrophin-Deficient Mice

**DOI:** 10.1371/4ffdff160de8b

**Published:** 2012-10-10

**Authors:** Daniel Brereton, Jeffrey Plochocki, Daniel An, Jeffrey Costas, Erin Simons

**Affiliations:** Arizona College of Osteopathic Medicine, Midwestern University; Department of Anatomy, Arizona College of Osteopathic Medicine, Midwestern University; Arizona College of Osteopathic Medicine, Midwestern University; Midwestern University; Department of Anatomy, Arizona College of Osteopathic Medicine, Midwestern University

## Abstract

The development of spinal curvature deformities is a hallmark of muscular dystrophy. While glucocorticoid treatment has been shown to prolong muscle function in dystrophic mice, its effects on the development of dystrophinopathic spinal deformation are poorly understood. In this study, we test the effects of glucocorticoid treatment on the onset of thoracolumbar kyphosis in the dystrophin-deficient mdx mouse using voluntary running exercise to exacerbate muscle fibrosis. We measure the kyphotic index, erector spinae muscle fibrosis, and vertebral bone histomorphometry in 4-month-old mdx mice in four groups: sedentary control, exercise-treated (continuous voluntary access to an activity wheel), glucocorticoid-treated, and glucocorticoid + exercise-treated. Exercise treated mice were found to have significantly lower kyphotic index (i.e., greater kyphosis) and greater muscle fibrosis relative to controls (p < 0.05). However, the deleterious effect of exercise on KI and muscle fibrosis was prevented by glucocorticoid treatment. Some differences in bone histological parameters were observed between treatment groups, suggesting there is a complex relationship between dystrophic muscular changes and vertebral bone mass. Our findings indicate glucocorticoid treatment delays the onset of thoracodorsal spinal deformation in mdx mice.

## INTRODUCTION

Mdx mice have a mutation in the gene that codes for dystrophin, an intracellular protein that anchors the actin cytoskeleton of striated muscle fibers to the extracellular matrix.^1^ ^2^ Dystrophin-deficient muscle fibers have a limited ability to stabilize the cell membrane during contraction and become necrotic over time, leading to progressive muscle wasting and fibrosis.^3^ ^4^ One consequence of dystrophinopathic muscle degeneration in both mdx mice and humans is the development of spinal deformities.^5^ ^6^ ^7^ ^8^ ^9^ In the mdx mouse, spinal deformation generally appears in the form of pathological thoracolumbar kyphosis after 4 months of age, and becomes significantly pronounced relative to control mice by 9 months of age.^7^ In humans, spinal deformities are a serious clinical concern because they contribute to respiratory dysfunction, the leading cause of death in Duchenne muscular dystrophy (DMD).^10^ ^11^

In this study, we test the effects of glucocorticoid treatment on the severity of thoracolumbar kyphosis in the mdx mouse. Current research suggests glucocorticoid treatment slows the progression of spinal deformation in humans, but it is unclear if it delays onset.^12^ ^13^ ^14^ The aim of our study is to determine if glucocorticoid treatment can be administered to delay the appearance of dystrophinopathic spinal deformation. We use voluntary exercise to exacerbate muscle fibrosis because mdx mouse dystrophinopathy is less severe than in DMD and therefore not a perfect model.^15^ Loss of muscle tissue and resulting muscle fibrosis is less pronounced in the mdx mouse because the muscle degeneration is not continuous, but is marked with intermittent periods of regeneration and reduced muscle fiber necrosis.^16^ This pattern allows the mdx mouse to retain muscle function longer in comparison with DMD patients. The use of voluntary exercise to exacerbate the symptoms of dystrophinopathy in the mdx mouse to more closely resemble DMD is common in studies of dystrophic pathophysiology and for pre-clinical testing of therapeutic interventions.^17^ ^18^ ^19^ We use 12-week-old mdx mice to test our hypothesis because voluntary running exercise has been shown to cause significant muscle deterioration at this age,^20^ yet pathological kyphosis is not normally present in mice this young.^7^ A treatment period of 4-weeks is used because long-term treatment with voluntary exercise (i.e., greater than 2 months) does not have the same deleterious effect in the mdx, and may even improve muscle function.^21^ ^22^ ^23^ Voluntary wheel running was preferred over treadmill running because it is less invasive, more cost-effective and time-effective, and yet still provides the desired results.

## MATERIALS AND METHODS

**Animals**

Forty mdx mice (C57BL/10ScSn-Dmd^mdx^, stock # 001801, Jackson Laboratory, Bar Harbor, ME) aged three months were randomly divided into a control sedentary group, exercise-treated group, glucocorticoid-treated group, and glucocorticoid + exercise-treated group (n = 10 per group). Mice in the exercise-treated group were allowed four weeks of continuous voluntary access to a rodent activity wheel. Running distance was monitored using magnetic counters that totaled the number of laps each mouse ran per day. Corticosteroid treatment consisted of twice-weekly injections of methylprednisolone (Thermo Fisher Scientific, USA) at a dose of 5 mg/kg for four weeks. Methylprednisolone was administered via intraperitoneal injection in a vehicle of 10% DMSO in 0.9% saline. Methylprednisolone doses ranging from 0.75 to 14 mg/kg have been shown to benefit muscle tissue in mdx mice.^24^ ^25^ ^26^ All mice were given a one-week period to acclimatize to their environment prior to the commencement of the 4-week study. Mice were housed individually and provided with food and water *ad libitum*. Animals were sacrificed after 4 weeks of treatment with compressed CO_2_ in strict accordance with the recommendations in *The Guide for the Care and Use of Laboratory Animals*, National Institutes of Health, Publ. No. 85-23, 1996. Use of animals was approved by the Institutional Animal Care and Use Committee at Midwestern University.

**Kyphotic Index**

Kyphotic severity was measured using the kyphotic index (KI). Following sacrifice, the lateral aspect of the abdomen and thorax was dissected away to reveal the vertebral column from approximately C7 to L6. The degree of thoracodorsal kyphosis was measured from digital images taken with a tripod-mounted camera [Nikon Coolpix P90] that was a fixed distance from the specimens. A scale bar was present in the field so that size could be accurately determined. KI was measured following the methodology described by Laws and Hoey [7], which was developed for assessment of KI in mdx mice. Mice were placed on a flat surface in right lateral recumbency with the right humeri and femora parallel to each other. Left humeri were abducted and flexed to expose the vertebral column. KI was calculated as the ratio of the length to depth of the kyphosis where length was the linear distance from the body of C7 to the body of L6 and depth was the maximum perpendicular distance from that line to the dorsal vertebral border (Fig.1). Smaller KI is indicative of greater kyphosis. Typical KI in control (C57BL/10ScSn) mice at this age is approximately 4.2.^7^ To ensure positioning of the mouse was consistent during the measuring of KI, a measurement repeatability analysis was conducted. During this blind analysis, each mouse was repositioned, imaged, and measured by a single observer five separate times. Repeatability was estimated using Dahlberg’s method error (ME) statistic by dividing the square root of the sum of squares between repeated measures by twice the sample size. The average ME was less than 0.1 mm for all measurements, indicating that measurement error related to positioning of the mouse contributed little to the variance of the sample.

**Kyphotic index (KI) was calculated as the ratio of length to depth of the thoracodorsal kyphosis.**
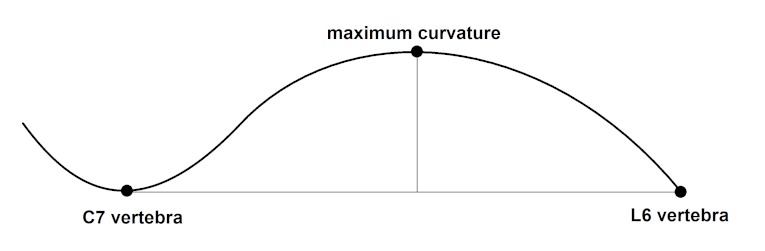
Length was measured as the linear distance from the ventral surface of the vertebral bodies of C7 to L6. Depth was measured as the maximum perpendicular distance from that line to the dorsal vertebral border.

**Muscle Histology**

The erector spinae muscle group was excised from the thoracolumbar region and immediately frozen in liquid nitrogen. Transverse cryosections were cut at a thickness of 12-µm at -20 C and stained with picrosirius red for fibrosis. Sections were digitally captured using an Eclipse 55i microscope (Nikon Inc.). Muscle fibrosis was quantified using the *Analyze Particles* function of the software ImageJ 1.42q (http://rsb.info.nih.gov/nih-image) after converting the images to 8-bit grayscale and adjusting the color threshold so only the stained regions were visible. To control for variation in muscle size among mice, the area of fibrosis was measured as a percentage of the total muscle cross-sectional area. Erector spinae muscles were chosen because of their postural and stabilizing roles during mouse running activity.

**Bone Histology**

Histomorphometric analysis of thoracic vertebrae was conducted to identify the effect of exercise and glucocorticoid treatment on the spine. The entire length of the vertebral column was harvested and cleaned of muscle tissue. Vertebral columns were then fixed in 70% ethanol for two days, dehydrated in graded alcohols, and embedded in methyl methacrylate (Polysciences, Warrington, PA). Vertebral columns were sectioned in the sagittal plane using a low speed saw (VC-50; Leco, St. Joseph, MI) and ground to a final thickness of 100-µm (Metaserv 250; Buehler, Lake Bluff, IL). Sections were stained with alizarin red for calcium and imaged using an Eclipse 55i microscope (Nikon Inc.). Measurements were taken from digital images of the T11, T12, and T13 vertebrae. These vertebrae were chosen because the maximum kyphosis was typically observed at the T12 vertebral level. Measurements included the area of bone tissue and the lengths and widths of the vertebral bodies of T11, T12, and T13.

**Statistics**

Statistical treatment of the data consisted of one-way ANOVA followed with Tukey’s HSD *post hoc* test. Kolmogorov-Smirnov tests and box plots were used to confirm that assumptions of the statistical treatments were not grossly violated. Data are presented as means ± standard error. Statistical significance was set at *P *< 0.05.

## RESULTS

KI was used to quantify the degree of thoracolumbar kyphosis in each mouse. Comparisons of KI among treatment groups are shown in Fig. 2 and spinal dissections are shown in Fig. 3. Mdx mice treated only with exercise had significantly lower KI (i.e., greater kyphosis) than sedentary mice (2.78 ± 0.08 and 3.50 ± 0.11, respectively; *p* < 0.01). However, the deleterious effect of exercise on KI was prevented by glucocorticoid treatment. KI of mice treated with glucocorticoid + exercise was significantly greater than that of exercise-treated mice (3.98 ± 0.18; *p* < 0.01), as was the KI of mice treated with glucocorticoid alone (3.85 ± 0.08; *p* < 0.01). No difference in KI was found between sedentary and glucocorticoid + exercise-treated mdx mice or between sedentary and glucocorticoid-treated mdx mice (*p* > 0.05).

**Comparison of KI among treatment groups.**
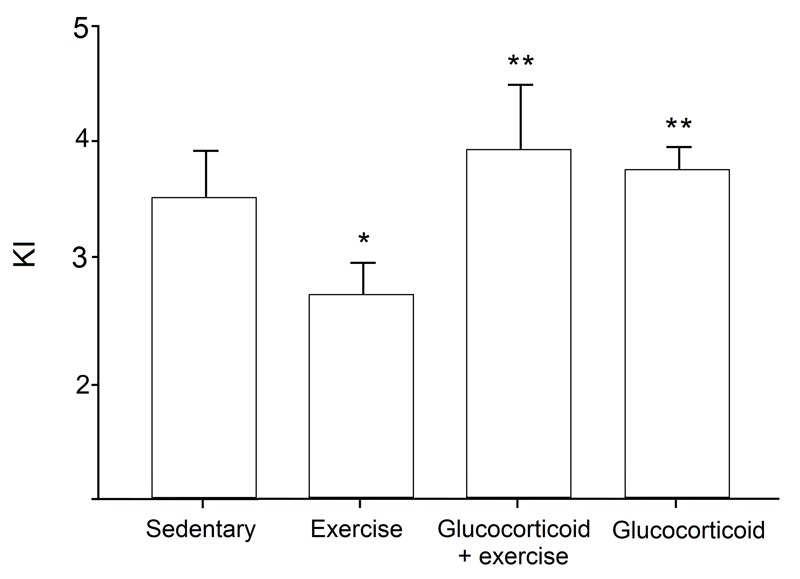
Exercise-treated mice had significantly lower KI (i.e., greater thoracodorsal kyphosis) than sedentary control mice. Mice treated with glucocorticoid + exercise and with glucocorticoid alone had significantly greater KI than exercised mice, but were similar to sedentary controls. * p < 0.05 compared with sedentary controls; ** p < 0.05 compared with exercised mice. Means ± 2SE.

**Dissections showing difference in KI.**
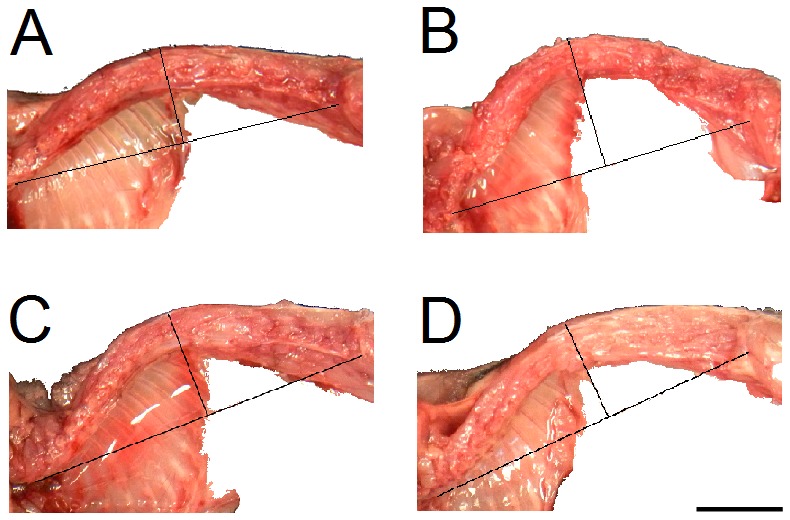
Images are of sedentary control (A), exercise-treated (B), glucocorticoid + exercise-treated (C), and glucocorticoid-treated (D) mdx mice. Exercise-treated mice exhibited significantly lower KI in comparison with all other treatment groups (p < 0.05). Scale bar is 1 cm.

**Fig. 4: Comparison of erector spinae muscle fibrosis among treatment groups.**
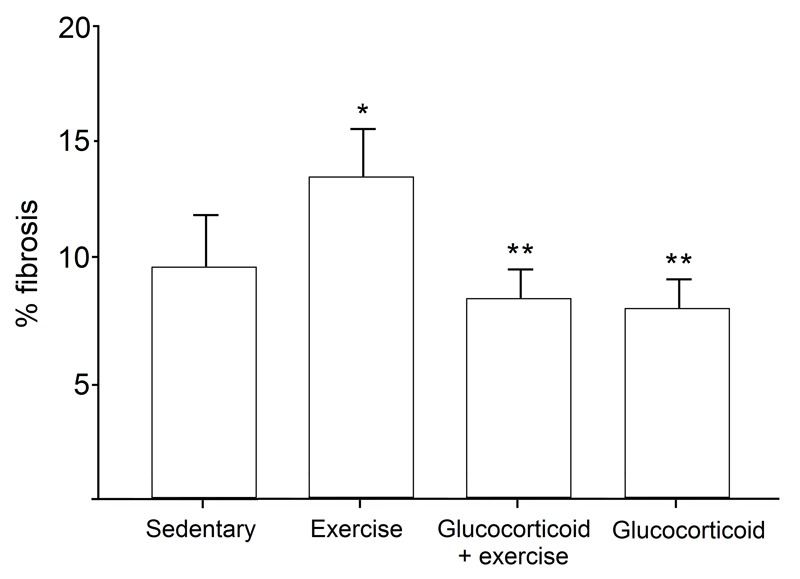
Erector spinae fibrosis was calculated as the percentage of fibrotic tissue relative to the total muscle area. Exercise-treated mice had significantly greater fibrosis in comparison to sedentary controls. Mice treated with glucocorticoid + exercise and with glucocorticoid alone had significantly less fibrosis than exercised mice, but were similar to sedentary controls. * p < 0.05 compared with sedentary controls; ** p < 0.05 compared with exercised mice. Means ± 2SE.

**Fig. 5: Photo micrographs of erector spinae muscle histological sections.**
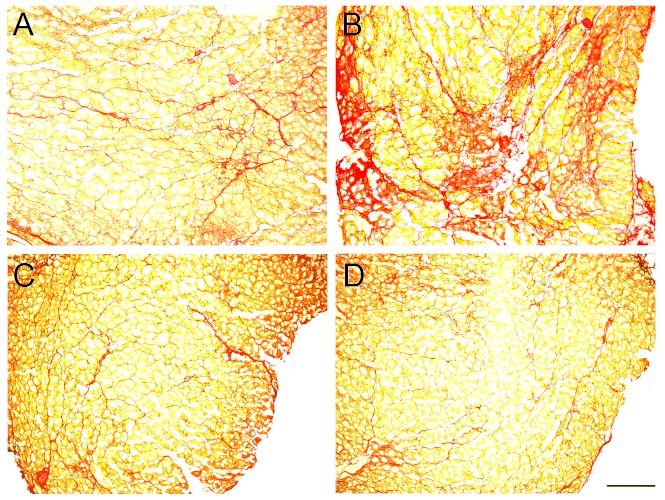
Images are of sedentary control (A), exercise-treated (B), glucocorticoid + exercise-treated (C), and glucocorticoid-treated (D) mdx mice. Mdx mice treated only with exercise exhibited significantly greater fibrosis in comparison with other treatment groups (p < 0.05). Scale bar is 100 µm. Picrosirius red, 40x magnification.

Comparisons of erector spinae muscle fibrosis, calculated as the percentage of total muscle area that was fibrotic (i.e., stained positively with picrosirius red), are depicted in Fig. 4. Representative histological sections of erector spinae muscles are shown in Fig. 5. Mice treated only with exercise had significantly larger amounts of fibrosis than mice in the sedentary control group (9.65% ± 1.12 and 13.79% ± 1.28, respectively; *p* < 0.05). Similar to KI comparisons, the deleterious effect of exercise on muscle fibrosis was ameliorated with glucocorticoid treatment. Mice treated with glucocorticoid + exercise had significantly less erector spinae fibrosis than exercised mice (7.75 ± 0.51; *p* < 0.01), as did mice treated with glucocorticoid alone (7.39% ± 0.75; *p* < 0.01). Again, no difference was found when comparing sedentary mice to either mice treated with glucocorticoid + exercise or glucocorticoid alone (*p* = 0.49 and *p* = 0.38, respectively).

Table 1 shows results from the analysis of bone histomorphometric parameters. Here, the greatest effect by treatment group was observed on the length and bone area of the T11 vertebra. Exercise-treated mice had significantly shorter T11 vertebrae in comparison to sedentary, glucocorticoid + exercise, and glucocorticoid-treated mdx mice (*p* < 0.05). T11 vertebrae of exercise-treated mice also had less bone area than glucocorticoid + exercise and glucocorticoid-treated mdx mice (*p* < 0.05), but were similar to sedentary controls (*p* > 0.05). Additionally, sedentary mice had significantly less bone area of the T13 vertebrae than mice treated with glucocorticoid alone (*p* < 0.05). No other differences in bone histomorphometry were found.

**Table:** 

**Table 1. Summary of bone histomorphometry from vertebrae T11-T13 by treatment group**				
	Sedentary (n=9)	Exercise (n=10)	Glucocorticoid + Exercise (n=8)	Glucocorticoid (n=9)
T11 length (mm)	2.12±0.16	1.73±0.04^ a^	1.87±0.07^ b^	1.86±0.08^ b^
T12 length (mm)	2.05±0.06	1.94±0.06	1.93±0.05	2.02±0.09
T13 length (mm)	2.11±0.10	2.24±0.08	1.96±0.16	2.26±0.12
T11 bone area (mm^2^)	0.66±0.06	0.52±0.08	0.68±0.06^ b^	0.63±0.04 ^b^
T12 bone area (mm^2^)	0.67±0.05	0.62±0.02	0.70±0.05	0.67±0.02
T13 bone area (mm^2^)	0.69±0.02	0.75±0.04	0.66±0.10	0.87±0.06^ a^
^a^ *p* < 0.05 versus sedentary; ^b^ *p* < 0.05 versus exercised				

To quantify voluntary running activity, average weekly running distances during the 4-week experiment were tabulated. Exercise-treated mice ran an average of 26.6 ± 4.5 km/week, while glucocorticoid + exercise-treated mice ran an average of 31.6 ± 2.9 km/ week. Running distance between these treatment groups was not significantly different (t = 0.934,* p* = 0.36 with student’s *t test*). Running activity remained consistent from week to week for each individual mouse, but the variance was high (σ^2^ = 145.5). Correlation between KI and muscle fibrosis in mice while controlling for running distance was significant (r_partial_ = -0.634,* p* < 0.01), indicating kyphosis becomes more severe (i.e., KI decreases) as erector spinae muscle fibrosis increases. KI was positively correlated with bone histomorphometric parameters (i.e., as KI decreased, bone lengths and areas decreased; multiple R^2^ = 0.322), while erector spinae fibrosis was negatively correlated with bone histomorphometric parameters (i.e., as muscle fibrosis increased, bone lengths and areas decreased; multiple R^2^ = -0.259).

## DISCUSSION

Consistent with previous findings, short-term voluntary running exercise exacerbated muscle fibrosis in mdx mice in our study.^17^ ^20^ ^27^ ^28^ Exercised mice had significantly greater erector spinae muscle fibrosis than sedentary control mice. Because functional decline of dystrophic striated muscle precedes muscle fibrosis in the mdx mouse,^29^ we can infer that significant impairment of erector spinae muscle function occurred in the exercised mice in our study. Muscle fibrosis was positively correlated with KI and negatively correlated with bone histomorphometric parameters. These findings further suggest increased muscle fibrosis leads to structural changes that affect spinal curvature, and are in agreement with other findings that spinal deformation is secondary to erector spinae muscle deterioration.^30^ ^31^ ^32^ Although the precise mechanism is unknown, previous studies show that striated muscle contraction from exercise increases the rate of necrosis of dystrophic myocytes, resulting in a decrease in maximal tetanic tension.^33^ ^34^ ^35^ Necrotic muscle tissue is then replaced with noncontractile connective tissue, further reducing muscle force. Such alterations in the mechanical environment of the spine affect vertebral bone mass and likely lead to kyphoscoliotic deformations.^7^ ^36^

Our data also support the hypothesis that glucocorticoid treatment delays the onset of spinal deformation in mdx mice. Mice treated with exercise developed severe thoracodorsal kyphosis several months earlier than is normal for the mdx mouse,^7^ while mice treated with glucocorticoid + exercise did not. Numerous studies have shown benefits of glucocorticoid treatment on dystrophic muscle function in the mdx mouse,^26^ but to our knowledge this is the first study that shows such treatment may delay its onset. Our findings suggest that reduced fibrosis in glucocorticoid-treated mice relative to exercise-treated mice is indicative of greater mechanical stability of the spine, which would inhibit spinal deformation. Mice treated with exercise alone exhibited significantly greater fibrosis than glucocorticoid-treated mice, suggesting a relative decrease in mechanical stability of the spine. However, further study is needed to determine the long-term benefits on glucocorticoid treatment on the development and progression of dystrophic spinal deformation. It has been shown that short-term steroid treatment improves muscle strength in mdx mice, but muscle strength in mdx mice exposed to long-term steroid treatment is no better than controls.^37^ Likewise, long-term glucocorticoid use in DMD patients has been shown to reduce bone mass.^38^ The effects of exercise and glucocorticoid treatment on vertebral bone length and area in our study were variable and location-dependent and may differ with treatment duration.

While our findings are of note, how they translate to the treatment of DMD remains unclear. The beneficial effects of glucocorticoids on both human and mdx mouse muscle tissue are well documented. Structurally, the vertebral geometry and trabecular morphology are similar in mice and humans,^39^ ^40^ ^41^ although mice have relatively smaller spinous processes and do not exhibit the degree of progressive increase in vertebral diameter caudally observed in humans.^42^ Biomechanically, mice exhibit a reduction in rotation at the lumbar spine compared to humans, although primary loading is from axial compression in both species.^40^ ^43^ Human and mouse vertebral bone is affected similarly by changes in the biomechanical environment.^40^ ^42^ However, skeletal deformation in mice related to neuromuscular weakness presents primarily as thoracodorsal kyphosis and not scoliosis because of differences in loading related to locomotor posture.^7^ Again, further study is needed to elucidate the precise relationship between the development of spinal deformation and glucocorticoid treatment in DMD.

## Competing Interests

The authors have declared that no competing interests exist.
